# Teaching research group leaders’ perceptions of their engagement in curriculum leadership

**DOI:** 10.3389/fpsyg.2022.944445

**Published:** 2022-09-26

**Authors:** Yiming Shan, Junjun Chen

**Affiliations:** ^1^School of Foreign Languages, Zhaoqing University, Zhaoqing, China; ^2^Department of Education Policy and Leadership, The Education University of Hong Kong, Tai Po, Hong Kong SAR, China

**Keywords:** curriculum leadership, teaching research group leader, decentralization, qualitative, China

## Abstract

Understanding how teacher leaders are engaged in curriculum affairs is critical with the implementation of instructional decentralization. The current study adopts a qualitative approach to investigate Teaching Research Group (TRG) leaders’ involvement in curriculum leadership (CL) in the Chinese context. It explores the conceptions of TRG leaders by interviewing 20 of them, observing four meetings held by TRG leaders, and collecting 10 extracts from appraisal summaries of TRG leaders in secondary schools in China. Drawing on the findings, this paper examines the characteristics of TRG leader’s engagement in CL. More importantly, data highlighted significant problems the participants perceived and faced in their work as TRG leaders, which consisted of amplifying the necessity for empowering TRG leaders and identifying the phenomenon that said leaders are less empowered and less motivated to undertake the CL role. The results add to the international body of knowledge on the teachers’ engagement in CL.

## Introduction

In recent decades, much educational reform has shone a focus onto the implementation of decentralization which transfers authority, responsibilities and tasks from the top down (Koh et al., 2014). This phenomenon enables schools and teachers to have more power and autonomy in curriculum issues and curriculum decision-making processes (Law et al., 2010). Meanwhile, much research attention has been paid to understanding curriculum leadership (CL) with the implementation of decentralization (Stark et al., 2002). However, research evidence related to issues of the teacher as leader in CL has just begun to emerge in the past 20 years, since a large body of the literature has focused on exploring the principal’s role in CL (Cummings, 2011; Jenkins and Pfeifer, 2012). Jorgensen (2016) noted that enacting CL is not only within the ambit of the principal but also within that of teachers. In recent years, teachers carry much more responsibility for curriculum matters (Wiles, 2009). However, studies related to teachers’ engagement in CL remain scant (Cummings, 2011; Ylimaki, 2011). According to the findings extracted from Hu and Gu’s (2012) commentary study, it is noted that few empirical studies of CL occurring in secondary schools in Asian contexts have been reported since Chinese literature mainly makes critical evaluation and commentary on western studies.

In China, the new curriculum reform (NCR) undertaken in 2001 engendered “major curriculum and instructional change” (Walker and Qian, 2012, p. 164). It involved efforts invested into conducting a policy of three-level (e.g., national, provincial, and school) curriculum management, which makes curriculum decentralization essential (Guo, 2013; Zeng and Zhou, 2013). Specifically, it is stipulated that the schools execute the national curriculum under the authoritative directive made by MoE and they are entitled to develop a school-based curriculum in accordance with their educational context (MoE, 2001). Such decentralization provides policy space for teachers to be granted more autonomy in curriculum matters (Wang and Zheng, 2013; Fu and Yu, 2014) and teachers’ roles to gradually change from followers to leaders in curriculum decision-making (Zhang, 2012; Zhong, 2013). As described by Wang and Zheng (2013), under the implementation of the NCR, it is the first time that teachers have assumed the roles of designers, developers, and leaders in curriculum development. However, it has been identified that teachers achieve only a low level of empowerment and have little influence on curriculum issues in Chinese schools (Chang and Li, 2007; Hu and Gu, 2012). Under such circumstances, examining CL in the Chinese context becomes a salient concern (Lu, 2011). Unfortunately, nonempirical studies still dominate the Chinese literature (Walker et al., 2012), and similar to the Western literature, the most relevant Chinese literature on CL has focused on identifying principals’ CL (Hu and Gu, 2012; Wang and Kang, 2013). Empirical studies investigating teacher leaders’ CL are scant (Long and Chen, 2010; Hu and Gu, 2012).

The Chinese Ministry of Education (MoE) defined the role of TRG leader as the one positioned between the principal, the director of studies, and teachers, and it emphasized that a TRG leader is not the director of administrative affairs (Price, 2005). Particularly, according to Guo’s (2007) statement, TRG leaders have never been recognized as the middle-level leaders although the responsibilities they assumed are quite related to the administrative affairs, such as arranging refresher courses for teachers, holding teaching competition and etc. On the other hand, it is identified that TRG leaders are backbone (*gugan*) teachers who possess management and leadership skills (Zhang, 2007). In the existing Chinese literature, it has been identified that teachers’ involvement in CL plays a pivotal role in improving teaching quality (Chen, 2014) and academic achievement (Xiong and Lim, 2015) and can also make up for limitations in the principal’s leadership (Dong, 2008; Mao, 2009). Thus, in light of these findings, a deeper understanding of how TRG leaders are empowered and enact CL is obviously significant. Most crucially, a need exists to develop and reconceptualize the knowledge base of teachers’ engagement in China to fill the research gap.

The focus of this research was on exploring how TRG leaders conceptualize their CL roles and analyzing challenges they encountered, thereby contributing to the literature related to teacher leaders’ engagement in CL. By analyzing the data, this study was able to identify key factors and difficulties in helping to empower teacher leaders, which can enable the development of a more cooperative school culture for the devolution of authority in curriculum matters.

## Literature review

### School leadership and CL

No authoritative definition of school leadership exists because it is a loosely defined term with various descriptions (Day and Antonakis, 2012). School leadership is regarded as a process that guides and influences individuals’ behavior (Yukl, 2006). Harris (2003) defines leadership as dynamic relations among stakeholders in learning organizations. According to the descriptions of Elliott et al. (1999, p. 174), leadership reflects the “role definition, power relationships and behavior of those who may be characterized as leaders.” In addition, Nashashibi and Watters (2003) depict leadership in detail as follows: (1) “leadership is a process of influencing others”; (2) “leadership can be exercised by people without formal authority or designation”; (3) “leadership implies that there are followers”; and (4) “leadership involves moving forward to achieve goals or objectives” (p. 48). In general, it appears that Elliot et al.’s (1999) definition reflects the functional traits and influential ability of school leadership that it is “a dynamic interplay of school-related factors and personal factors” that assists the achievement of school goals (Elliott et al., 1999, p. 171).

The exercise of leadership plays a key role in school development because it affects the success or failure of school development (Hallinger and Heck, 2010; Day and Sammons, 2013) and also the school culture (Harris, 2003). Leadership has been confirmed to directly affect students’ academic achievement, thereby facilitating students’ learning (Lee et al., 2012; Walker et al., 2014). Furthermore, the enactment of leadership contributes to building staff capacity and instructional ability, such as motivations, values, commitments, competence, and a knowledge base in teaching (Day and Sammons, 2013; Whitworth and Chiu, 2015).

In the field of leadership, CL has been given increasing attention since some studies began to explore schooling issues through reviewing curriculum implementation and curriculum development (Macpherson et al., 1998; Ylimaki, 2012). According to Bush and Glover’s (2014) description, CL is viewed as a type, an alternative or a model of school leadership which is used to denote a focus on leadership concerned with managing teaching and learning activities.

No commonly agreed definition of CL exists (Tsui, 2014). It is regarded as “an amorphous role” that falls to “a person or group of people who assume responsibility for curriculum” (Jorgensen, 2016, p. 370), such as principals, administrators, and teachers (Macpherson and Brooker, 2000). It is also defined by its functions, which include taking administrative duties (Nashashibi and Watters, 2003), instructional responsibilities, or any initiative related to “the sociocultural and political aspects of educational content” (Ylimaki, 2012, p. 305). Furthermore, the exercise of CL is described as the interplay between the leader and other stakeholders in schools, community, and society (Ylimaki, 2011). In addition, some researchers have presented that the enactment of CL means taking initiatives in a specific context, namely at “macro- (e.g., starting a new school) and micro-levels (e.g., implementation of curriculum policy in a single class)” (Macpherson et al., 1998, p. 76). Moreover, the enactment of CL also reflects personal qualities and professional identities, such as the knowledge, beliefs, self-awareness, values, and experiences that the individual brings to the organizational context (Ylimaki, 2011). In general, through integrating the aforementioned descriptions, it seems logical to define CL from four perspectives: building vision for curriculum development at the school level, coordinating curriculum at the classroom level, communicating curriculum issues at the social relationship level, and reflecting individual’s professionalism and qualities at the personal level.

The enactment of CL plays a pivotal role in creating “positive learning and safe, orderly schools” (Ylimaki and Brunner, 2011, p. 1264). In classroom settings, exercising CL provides positive reinforcement for students learning (Handler, 2010) and influences students’ learning experiences (Xiong and Lim, 2015). As for the social relationship level, CL has been assumed to prompt teacher collaborations, and thus ultimately prompt staff development and the formation of cooperative school climate (e.g., Copland and Knapp, 2006; Law and Wan, 2006). Studies have also identified that individuals who are equipped to enact CL are required to have professional skills, specialized knowledge, competencies in curriculum, communicative ability, and even an understanding of psychology, thereby further enhancing personal development (e.g., Chval et al., 2010; Cummings, 2011).

### Role of teacher leaders in CL

Teacher leaders are the type of leaders who exercise “strong professional-oriented leadership practices” (Chen and Zhang, 2022, p. 1). According to Gao and Hu’s (2016) statement, backbone teachers, subject heads, and TRG leaders are all belongs to teacher leaders. TRG leaders are appointed to take charge of curriculum matters in their subject area. The MoE first named the role of TRG leader (*jiaoyan zuzhang*) in the Secondary School Teaching Research Group Rulebook (draft) in 1957.

Some scholars have described TRG leaders as TRG heads (Qian et al., 2016) or the head of TRG (Yuan et al., 2018). To some extent, a TRG leader is equivalent to various terminologies such as the head of department (HoD) in the British context (Li and Edwards, 2014), subject leader (also in the United Kingdom), or chair (in the United States). However, in the Western context, the HoD is regarded as an academic middle manager (Mercer and Ri, 2006; Dinham, 2007), whereas in China, TRG leaders are not middle-level leaders in schools (Guo, 2007; Li, 2013). Notably, the HoD in the Western context is a middle manager who has formal responsibilities and roles such as teaching, learning, and the curriculum; monitoring, evaluating, and improving people and relationships; and managing resources and accountability (Adey, 2000). In Chinese secondary schools, however, these discrete roles are split up (Mercer and Ri, 2006). As Mercer and Ri (2006, p. 107) indicated, the HoD is “a different creature” in the Western context, whereas “there is not the same level of interest in the role of the HoD in Chinese secondary schools.” The obvious difference between a TRG leader and a HoD was identified as being that a TRG leader is not the director responsible for all administrative affairs in the school’s management system (Price, 2005).Although no authoritative definition exists of TRG leader, Chinese researchers have defined the role and responsibilities of a TRG leader. It has been noted that TRG leaders are outstanding frontline teachers (Gao and Hu, 2016), organizers (Chen, 2014), or subject leaders (Du, 2013). Furthermore, Zhang (2012) articulated that a TRG leader is the core leader of a TRG building, a demonstrator of teaching, the backbone of the improvement of teaching quality, the bellwether of research, and the implementer of teacher development programs. Furthermore, other researchers have outlined TRG leaders’ responsibilities, such as building a shared vision, promoting the organizational culture, sharing teaching experiences, developing teaching beliefs, guiding research, organizing group activities, and promoting group members’ continual professional development (Chen, 2014).

In conclusion, TRG leaders are curriculum leaders who assumed to undertake pedagogical considerations for teaching and research matters, and lead teachers of the same subject to collaborate. They are the frontline teachers and backbone (*gugan*) teachers who retain power on the part of middle managers but are not middle managers.

### Teachers’ involvement in CL

With the implementation of educational decentralization, teacher involvement in the decision-making process for curriculum matters has “long” been the focus of research interest (Ho, 2010, p. 613).

At the school level, teachers are empowered with autonomy in making administrative and instructional decisions, such as cultivating school culture and climate (Macpherson et al., 1996; Wang, 2013); building a shared vision and setting goals for curriculum development (DeMatthews, 2014); allocating teaching sources (Lin and Lee, 2013; Wang and Kang, 2013); and providing instructional suggestions (Luo and Xia, 2011). Researchers have identified that teacher involvement in CL improves a school’s security and stability (Ylimaki and Brunner, 2011); enhances a democratic, open, and collaborative school culture (Luo and Xia, 2011); and thus finally prompts school development (Dong, 2008; Campbell and Malkus, 2011). However, teachers are not fully involved in taking on the CL role in the authentic context because of the rigid organizational structure and undemocratic schooling (Wang and Zheng, 2013; Moreeng and Tshelane, 2014).

At the classroom level, teachers who assume CL are responsible for setting curriculum goals and spearheading teaching resources for ensuring compliance with curriculum standards (Cummings, 2011; Fu and Yu, 2014), and also devoting to classroom initiatives such as solving teaching and learning problems as well as organizing classroom activities for effective teaching and learning (Macpherson and Brooker, 2000; Law et al., 2007). Furthermore, these teacher leaders are in charge of making assessments of teaching and evaluating learning, and also monitoring the curriculum implementation (Huang and Zhu, 2015; Xiong and Lim, 2015). The literature also confirms that teachers who served in CL positions have an effect on enhancing teaching quality (Cummings, 2011; Luo and Xia, 2011) and improving academic achievement (Law and Wan, 2006; DeMatthews, 2014), thereby ultimately prompting school improvement (Wiles, 2009) as well as the implementation of curriculum reform (Wang and Zheng, 2013). However, teacher leaders’ autonomy in curriculum decision making is low because it is normally constrained by curriculum standards (Macpherson and Brooker, 2000; Zheng and Guo, 2010).

At the social relationship level, teachers’ involvement in CL mainly involves three aspects. That is, sustaining relations with superiors (e.g., principals and deputy principals) to convey problems or obtain strategic direction for school-wide programs (Wu, 2003; Chval et al., 2010); with subordinates to prompt collaboration and solve problems on teaching and research (Ye and Zhu, 2013; Albashiry et al., 2016); and with external stakeholders (e.g., other schools or district administrators) to communicate and share information and experiences (Wang and Kang, 2013; Albashiry et al., 2016). Maintaining relations with stakeholders plays a crucial role in school development (e.g., Gabriel and Farmer, 2009). Teacher involvement in CL can compensate for deficiencies in the principal’s leadership (Mao, 2009). By contrast, collaboration among teachers and between schools prompts experience-sharing and individual professional development, which builds a positive school culture (Nashashibi and Watters, 2003; Li and Duan, 2004). In the real context, however, maintaining relationships with stakeholders entails challenges, such as insufficient support from principals (Dong, 2008; Chval et al., 2010), an uncooperative atmosphere among peers (Fu and Yu, 2014), and less communication with stakeholders outside schools (Zhang and Xie, 2012).

At the individual level, CL has been assumed to involve maintaining awareness of being empowered to be leaders (Macpherson et al., 1996; Xiong et al., 2011); being equipped with knowledge and skills such as curriculum design, curriculum implementation, curriculum evaluation, and educational theories and policy (e.g., Handler, 2010; Ye and Zhu, 2013); and possessing professional ethics such as devotion (Wang and Zheng, 2013), risk taking (Li, 2004), sharing (Ye and Zhu, 2013), and trustworthiness (Zheng, 2007). Evidence from a relevant study identified that teacher leaders who possess awareness, knowledge, skills, as well as professional ethics affect the success of individuals and organizations (Nashashibi and Watters, 2003). Nevertheless, teacher leaders demonstrate little desire and even less ambition to take on the CL role (Handler, 2010), and some teachers’ professionalism is relatively limited (Mabry and Ettinger, 1999; Wang, 2013). In particular, some teachers in the Chinese context still lack a sense of responsibility (Lin and Feng, 2007), and they are selfish and utilitarian (Ye and Zhu, 2013), which results in their inactiveness toward taking on the CL role.

In conclusion, although research on CL in the Chinese context exists, there is a dearth of research on TRG leaders’ CL role, especially research with empirical data (e.g., Wang and Kang, 2013). Furthermore, most earlier studies are structured to critically evaluate findings emanating from Western studies, and thus their arguments are based on insufficient Chinese empirical data (Hu and Gu, 2012). Thus, an in-depth understanding of TRG leaders’ engagement in CL in the Chinese context may still be required. A qualitative study was deployed to explore TRG leaders’ perceptions of said leaders’ engagement in CL in secondary schools in mainland China. This study intended to answer two main research questions (RQs):

(RQ1) What are the characteristics of TRG leaders’ engagement in CL?
(RQ2) What challenges arise when empowering TRG leaders?

## Materials and methods

In order to examine TRG leaders’ in-depth perceptions of how TRG leaders are engaged in enacting CL roles, the intepretivism approach was adopted in a bid to extract a rich set of data “based on people’s experiences and their understanding of them” (Gemma, 2018, p. 8).

### Participants

In this research, a purposive sampling technique was used for data collection. This deliberated choice of participants enables the researcher to identify and obtain rich information related to the research topic (Elo et al., 2014). Because few empirical studies on teachers’ CL have been conducted in secondary schools in Asian contexts (Hu and Gu, 2012), and the implementation of NCR has mainly targeted the secondary school context (Tang et al., 2011), the researcher thus attempted to collect data in secondary schools, China. Under the policy of instructional decentralization, schools are entitled to autonomy in curriculum matters. Thus, with the aim of enhancing the representativeness of the results, the researched schools were selected on the basis of the current pattern of education facilities in China and all school types were covered (see Table 1).

**Table 1:** School type.

School characteristics			*n*
State-run schools	Key schools	Provincial key school	1
		City/Local key schools	4
	Non-key schools	Ordinary schools	4
Private school			1

S, School.

A researcher identified that samples of 12 should be adequate for exploring participants’ perceptions (Boddy and Boddy, 2016). In this research, to ensure representative balance, 20 participants were TRG leaders who taught different subjects and held various lengths of work experience in the role of TRG leader. In particular, their gender, experience of being TRG leader, and teaching subject were collected (see Table 2). For respecting the rights and dignity of participants (Oates et al., 2010), the ethical approval of this study was obtained through sending the consent form to the schools and participants before its commencement.

**Table 2:** TRG leaders’ demographic information.

Code	Gender		Experience of being a TRG leader			Teaching subject	
	Male	Female	>10 years	5–10 years	<5 years	Science	Humanities
TRGL1		√	√			√	
TRGL2		√		√		√	
TRGL3		√			√	√	
TRGL4		√		√			√
TRGL5		√			√	√	
TRGL6	√		√				√
TRGL7		√		√			√
TRGL8		√			√		√
TRGL9	√		√			√	
TRGL10	√		√			√	
TRGL11		√		√			√
TRGL12		√	√			√	
TRGL13	√		√				√
TRGL14		√		√			√
TRGL15		√		√		√	
TRGL16		√		√			√
TRGL17		√		√		√	
TRGL18		√	√				√
TRGL19	√				√	√	
TRGL20		√			√		√
n	5	15	7	8	5	10	10

TRGL, TRG leader.

### Data collection

Three data collection techniques were deployed in this study to buttress one another. First, the semistructured interview technique was adopted to explore more hidden and in-depth information from respondents (Kvale and Brinkmann, 2009). Each interview lasted approximately 40 min, was audio-recorded, and was then transcribed verbatim for content analysis. Questions were designed based on a theoretical framework related to TRG leaders’ engagement in CL and were revised based on the pilot study. Issues explored with TRG leaders included participants’ demographic information, perceptions of TRG leaders’ engagement in CL as characteristics of enacting CL, significance of enacting CL, and challenges of enacting CL. For eliciting more insights and understanding in interviews (Emerson et al., 2011), field notes were taken after interviews to help memorize key points that emerged in interviews.

Second, observations of meetings were taken to obtain access to the authentic context and uncover relations and interactions among participants (Rubin and Rubin, 2012). In particular, four types of meetings held by TRG leaders were observed and video-recorded, which included the one held at the beginning of the semester for making the work arrangement and three monthly meetings for routine issues, team building issues, and teacher development issues, respectively.

For data triangulation, documents containing 10 extracts of TRG leaders’ performance summaries were obtained and studied to verify information that could not be observed (Patton, 2015).

### Data analysis

Qualitative data from the interviews, meeting observations, and documents were systematically analyzed. To obtain in-depth meanings, content analysis of data was employed for various sorts of data (Schreier, 2012). The data analysis process involved three phases. First, coding categories were established based on the literature and RQs. The categories and samples of quotes are displayed in Table 3.

**Table 3:** Sample of data coding outputs.

Category	Subcategories	Sample quotes
Enact CL at the School Level	Characteristics	*We make adjustment under the macro-control of the schooling system. (TRGL6)*
	Difficulties	*There exists a hierarchy in the school’s management system. TRG leaders cannot make autonomous decisions in that they must follow the guidance of the three-level curriculum management system. (TRGL16)*
Enact CL at the Classroom Level	Characteristics	*TRG leaders arranged teachers to observe a 45-min class and then make a weekly class evaluation. (MO3)*
	Difficulties	*We cannot make autonomous decisions for the national curriculum. (TRGL16)*
Enact CL at the Social Relationship Level	Characteristics	*TRG leaders coached and mentored young teachers for making preparations for Teaching Competition. (MO4)*
	Difficulties	*I feel tired to communicate and assign tasks to teachers. Young teachers have procrastination. Elderly teachers are inactive. (TRGL 5)*
Enact CL at the Personal Level	Characteristics	*TRG leaders are required to be responsible for taking on the CL role. (DPS7)*
	Difficulties	*I do not want to waste the time and energy in structuring how to implement the leadership practices. (TRGL 15)*

MO, meeting observation; DPS, document of performance summary; TRGL, TRG leader.

Subsequently, to ensure coding reliability, a peer review was conducted by an individual possessing a doctoral degree in educational leadership for testing the accuracy of the coding categories and the coding scheme, who then met with the author to compare codes. When there was no agreement on codes, data were reread and discussed until clarity and consistency were reached. As a result, a Kappa value of 0.85 was achieved through comparisons of coded transcripts, which can be regarded as satisfactory because Krippendorff’s alpha (Kalpha >0.70) shows the standard reliability statistic for content analysis (Krippendorff, 2013).

Data were then systematically coded and analyzed using the NVivo 11, which was employed to facilitate qualitative data analysis through browsing, manipulating, coding, and interpreting (Azeem et al., 2012).

## Findings

### General conceptions: Being unfamiliar with the term CL

Notably, 13 TRG leaders (65%) did not know or had not heard of the concept of CL unless the term CL was paraphrased into curriculum matters that they could provide some descriptions of. Evidence from the interviews indicated that CL refers to taking initiatives related to educational concerns such as designing course construction (TRGL9), taking in-class initiatives (TRGL2), and leading research projects on curriculum issues (TRGL8). In particular, enacting CL involves taking instructional initiatives for both the national curriculum and school-based curriculum. TRGL9 stated the following:

We follow the national curriculum standards when taking the national curriculum. Meanwhile, we develop our own characteristics for the school-based curriculum. I think implementing CL is a combination of particularity and universality. (TRGL9)

In general, although TRG leaders depicted curriculum issues in various manners, they were not entirely familiar with the expression of CL.

### The CL practices of TRG leaders

#### Taking instructional initiatives at school and classroom level

Findings of TRG leaders’ engagement in CL for the national curriculum and school-based curriculum were quite different.

Concerning TRG leaders’ involvement in CL for the national curriculum, all TRG leaders acknowledged that they cannot make any autonomous decision for instructional issues such as teaching hours, teaching contents, and plan of instruction, because these initiatives are restricted by the policy of the national curriculum standards. TRGL7 noted that,

Although we are appointed as the TRG leaders to be in charge of curriculum matters, we have no power in making decisions or any changes to the national curriculum. All the instructional decisions must be strictly in accordance with the guidance and requirements stipulated in the national curriculum. (TRGL7)

In particular, 15 TRG leaders (75%) emphasized that there is no autonomy over textbook selection. TRGL15 stated the process of textbook selection as follows:

There is a booklist specified by the MoE that strictly requires the local bureau of education to select the textbook from the list. Textbooks listed by the MoE are in conformity with the national curriculum requirements and approved by the State Textbook Examination and Approval Committee. Thus, schools have no autonomy to choose the textbooks, let alone teachers themselves. (TRGL15)

Participants ascribed this phenomenon to the High School Entrance Examination and College Entrance Examination. TRGL6 explained,

All the teaching content in the textbooks recommended by the MoE is involved in the examination scope. Teaching serves the examinations. It is necessary to use the appointed teaching materials, since most students take the national entrance examination. (TRGL6)

Different from the situations at school level, 13 TRG leaders (65%) acknowledged that TRG leaders can make some decisions in classroom teaching for the national curriculum. Evidence from the documents of, for example, TRG leaders’ performance summaries revealed that TRG leaders can make decisions when choosing what teaching approach to employ and adjusting their teaching schedule according to students’ learning effectiveness and teachers’ reflection on teaching (e.g., DPS2, DPS8). In addition, for obtaining enhanced teaching performance and learning outcomes, TRG leaders take responsibilities for conducting teaching and researching activities such as holding seminars on curriculum reform (e.g., TRGL2, TRGL3), analyzing the policy of curriculum standards or examinations (e.g., TRGL1, TRGL4), sharing teaching experiences (e.g., TRGL11, TRGL15), organizing peer class observation, and making reflections for teaching (e.g., TRGL9, TRGL17). Although TRG leaders have limited and restricted power in taking on the CL role for the national curriculum, seven TRG leaders (35%) indicated that teachers who participate in CL played a significant role in school development. TRGL17 explained as follows:

Teaching and researching are the core of the foundation of school development. TRG leaders’ engagement in CL is the mainstay of teaching and researching. Therefore, the development of a school has a close relationship with the implementation of CL. (TRGL17)

In general, the findings indicated that TRG leaders have less autonomy in the national curriculum because the initiatives are restricted by the national curriculum standards.

On the other hand, in terms of TRG leaders’ involvement in CL for the school-based curriculum, all TRG leaders indicated that they took more responsibility for curriculum matters because there were no unified curriculum standards for the school-based curriculum. As TRGL5 described,

We can take macro control over issues related to goal setting and goal planning on teaching issues or curriculum issues at the school level. (TRGL5)

Moreover, evidence from observations at, for instance, the monthly meeting for organizing routine issues (MO2) indicated that TRG leaders can make autonomous decisions for the school-based curriculum at the school level, such as discussing the course setting and working out a plan for the collective lesson preparation.

Furthermore, all TRG leaders acknowledged that they had relatively more autonomy in decision-making in classroom settings, which involves choosing the teaching content, making and adjusting instruction plans, and tailoring teaching materials. TRGL10 added that,

We can make decisions on what to teach and how to teach according to the students’ needs and ability. It is flexible to adjust the teaching schedule and there is no need to follow the unified curriculum standards for the school-based curriculum. (TRGL10)

TRGL6 offered an example of how a teacher leader enacts the CL role:

When the teachers decided to open the Literary Appreciation course, we discussed the feasibility of opening this course within the TRG. For the teaching content, for example, we added classical literature such as Tao Te Ching to this course since it could broaden students’ knowledge base of Chinese literature. And the added teaching content is not illustrated in the national curriculum teaching materials. (TRGL6)

In the same vein, TRG leaders, who are also the frontline teachers, undertake more responsibilities and are empowered with much autonomy in classroom teaching. Evidence from the interviews demonstrated that TRG leaders can decide many instructional issues, such as what knowledge point should be taught first and what should be second (TRGL8), what contents should be added or deleted to suit students’ needs (TRGL 16), and how to control the pace of teaching (TRGL7). In addition, TRG leaders are responsible for conducting teaching and research activities after their class teaching. Furthermore, evidence from the performance summary documents (DPS7, DPS10) and meeting observations (MO3) revealed that TRG leaders are responsible for arranging teachers from the TRG to observe peers’ classes and hold weekly post-evaluation meetings for reflecting, sharing teaching experiences, and solving problems in teaching. However, a key challenge facing TRG leaders when enacting CL for both the national curriculum and school-based curriculum is—as 14 TRG leaders (70%) noted—that no unified criteria exist for them to use as a reference when making curriculum evaluations. TRGL7 indicated,

The criteria for evaluation are based on our knowledge and experiences. There are no specific or unified criteria for curriculum evaluation. (TRGL7)

Although the TRG leaders still had to face difficulties, six of them (30%) emphasized that being empowered with the autonomy to organize and conduct teaching and researching activities would hold the key for school and individual development, and ultimately promote students’ learning.

It could be concluded from the TRG leaders’ perceptions that TRG leaders have extensive autonomy in making decisions for the school-based curriculum.

#### Nurturing relationships at the social relationship level

This study identified from the data that nurturing and maintaining relationships with superiors, subordinates, as well as stakeholders outside the school are crucial.

First, as evidenced from the interviews, 13 TRG leaders (65%) showed that TRG leaders always communicate with the deputy principals who are in charge of teaching affairs. TRG leaders not only communicate on teaching issues but also on unrelated teaching issues. The following excerpt is from TRGL10:

I usually communicate with the deputy principal rather than the principal, since deputy principals are responsible for teaching matters. We always get orders from the deputy principal at the beginning and the end of the semester, and before the mid-term examination and final examination for making arrangements and preparations in advance. Besides, we discuss the issue of awarding teachers who have excellent teaching performance at the end of the semester. (TRGL10)

In addition, 10 TRG leaders (50%) stated that they have close connections with the school’s Office of Academic Affairs on curriculum matters and with the school’s Teaching and Research Center on teaching on research issues. As TRGL3 reflected, TRGL5 also described the connections with the Teaching and Research Center as follows:

The Teaching and Research Center conveys information to TRG leaders, such as information about teacher training and seminars, or the educational documents and requirements made by the local Teaching and Research Center. At the beginning of the semester, the Teaching and Research Center gives us the teaching and research objectives. Also, we give a summary report of the accomplishment of objectives at the end of the semester. (TRGL5)

Second, TRG leaders are engaged in cultivating collaborative relations with subordinates through providing guidance on teaching and research, such as organizing collective lesson preparation (TRGL12), arranging lesson demonstration and class observation (TRGL11), and designing examination papers (TRGL8). Furthermore, 14 TRG leaders (70%) reflected that TRG leaders are in charge of organizing professional-development initiatives with teachers. Evidence from the meeting observations (MO3) demonstrated that the TRG leaders hold post-evaluation meetings to reflect and solve problems that emerged during class observation, and also arrange experienced teachers to share teaching experiences, new teaching approaches, and teaching sources with peers. Furthermore, in MO4, the TRG leader also encouraged teachers to participate in the Teaching Competition and promised to offer supportive assistance. TRGL14 mentioned,

TRG leaders learn new things (e.g., flipped classroom, microlectures) in the middle-level training first, then share with teachers in the teaching and research activities. (TRGL14)

Moreover, TRG leaders are responsible for nurturing relations within the groups. Evidence from the performance summary documents (DPS5) showed that the TRG leaders have been engaging in coordinating and building positive and collaborative relationships between the leaders of the lesson preparation group and teachers, with the aim of building a harmonious climate and strengthening rigorous academic attitudes.

Third, findings demonstrated that TRG leaders are also in charge of communicating with stakeholders outside the school; 19 TRG leaders’ (95%) acknowledged that the most frequent connecting channel is the local educational bureau, which holds middle-level training sessions every year for TRG leaders. The TRG leaders mentioned that the training is mainly related to teaching topics such as an introduction to flipped classroom teaching and micro-classes. TRGL20 stated,

There is a chat group set up by the local Teaching and Research Center for announcing issues related to teaching and research activities or middle-level training. Personally, I have no channel and I seldom connect with other learning organizations. (TRGL20)

However, in actual fact, several obstacles are encountered by the TRG leaders when they maintain relations with stakeholders in and outside the school. Half of the TRG leaders (50%) acknowledged that they receive insufficient support from the principals, which makes them dissatisfied and inactive when enacting CL. TRGL2 complained,

Being the TRG leader is a thankless job and we seldom get inspired or even any verbal praise from superiors. (TRGL2)

The findings led to the inference that the TRG leaders were dissatisfied with the status quo. Furthermore, eight of them (40%) complained that they received pressure from their principals. TRGL17 expressed her tensions as follows:

Because of the bureaucratic hierarchy, we mostly follow the principal’s orders rather than reflect issues to him. We seldom get inspired from superiors. Interestingly, if you report too much, the principal will question your competence. (TRGL17)

On the other hand, the climate in groups is rather unmotivated; 14 TRG leaders (70%) noted that some teachers, especially the young and elderly ones, are unmotivated, which brings stress and difficulties in implementing CL. One of the TRG leaders expressed her complaints as indicated in the following quote:

Millennials are independent and assertive. They do not want to be constrained. Thus, communicating with them makes me feel tired, since when I assign tasks to teachers, they are inactive. Also, young teachers procrastinate if you do not push them. (TRGL3)

Moreover, some TRG leaders acknowledged that leading elderly teachers to take part in the teaching and research activities is not easy. TRGL5 explained,

Some elderly teachers do not want to undertake duties since they are not interested in new things and want to be stable. (TRGL5)

Moreover, three TRG leaders (15%) complained that they get pressured. TRGL20 stated the following:

The local Teaching and Research Center holds middle-level training for TRG leaders. This should be a good action, but the organizers of the Teaching and Research Center strictly control the attendance records, and give us assignments such as writing teacher reports of continual professional development or giving suggestions on classroom teaching reform. These requirements bring pressures and increase our burden. (TRGL20)

In summary, the aforementioned results indicated that TRG leaders are involved in sustaining relationships with stakeholders when undertaking the CL role. However, they rather struggle with the uncooperative climate within the TRG.

#### Demonstrating capacity of enacting CL at The personal level

In identifying TRG leaders’ capacity, there are two aspects that are in accordance with said leaders’ different roles in enacting CL. On the one hand, regarding being the leader in the TRG, some TRG leaders (three, 15%) were identified as having an awareness of taking on the CL role and recognizing the importance and necessity for TRG leaders to build up awareness in taking on the CL role. For example, TRGL9 addressed this as follows:

Being a TRG leader of the PE Group, I always actively lead and organize the group to participate in competitions to broaden teachers’ knowledge of teaching and practice. Meanwhile, I have been pursuing professional development, which enables me to lead my group and my teachers. (TRGL9)

On the other hand, being the frontline teachers, TRG leaders are always experienced in teaching and are the backbones of team development. TRG leaders recognize the need to provide role models for teachers, as described by TRGL5:

Being the TRG leader, I must set a good example for peer teachers. I must push myself to learn new thing since knowledge is infinite. The premise of taking on the CL role is to be equipped with the foresight for curriculum development and with extensive knowledge and experience in teaching. (TRGL5)

In the findings, 16 TRG leaders (80%) acknowledged that TRG leaders’ professionalism must be sufficiently strong enough to convince other teachers and provide support for them. The TRG leaders indicated that they should be capable of rich teaching experiences (TRGL6, TRGL13), outstanding research ability (TRGL15), or foresight for the subject area (TRGL8).

Furthermore, evidence from both the interviews and documents demonstrated that TRG leaders should possess professional ethics, such as persistence when facing difficulties (TRGL9, TRGL11, TRGL13, TRGL17), responsibleness for taking on the CL role (TRGL19, TRGL20), patience when facing misunderstandings from superiors or subordinates (TRGL8, TRGL20), enthusiasm about enacting CL (TRGL18), and fairness when evaluating teachers’ performance (TRGL1, TRGL15). Furthermore, as shown in the performance summary documents, TRG leaders were required to be responsible (DPS2, DPS7).

However, TRG leaders are faced with two major obstacles when they enact CL. First, all TRG leaders interviewed acknowledged that TRG leaders had a lack of awareness in taking on the CL role. 12 TRG leaders (60%) admitted that they are inactive in taking initiatives for enacting CL. Furthermore, TRGL7 said that,

I seldom take initiatives actively without getting orders from the superior department. For one thing, I do not want to bother the superiors, for another I do not want to bring trouble to myself. (TRGL7)

Particularly, nine TRG leaders (45%) expressed confusion about the role of CL. TRG leaders face several problems, such as being unclear about their autonomy and power when enacting CL (e.g., TRGL6, TRGL19, TRGL20), being unsure of their leadership roles (e.g., TRGL4, TRGL18), or not believing in the importance of their engagement in CL (e.g., TRGL5, TRGL12).

In addition, five TRG leaders (25%) expressed unwillingness to be given more power. Participants indicated that they prefer following orders assigned by their superiors. TRGL15 said,

I like being led rather than leading. I just want to put all my energy and efforts into teaching and do not want to waste energy in structuring how to lead. (TRGL15)

Second, seven TRG leaders (35%) acknowledged that most TRG leaders do not possess management and communication skills relating to how to enact CL, although they are empowered with some autonomy in taking initiatives. TRGL9 stated,

I experience difficulty and feel helpless when taking on CL since I was not trained to be a leader and I lack related skills and experiences of how to enact CL. (TRGL9)

Some participants provided explanations that TRG leaders seldom or never take training related to cultivating management skills or communication skills. As noted by TRGL17,

Training for TRG leaders is organized by the local Teaching and Research Center. However, this training focuses on cultivating teachers’ ability in teaching rather than skills or knowledge related to taking on the leadership role. (TRGL17)

The aforementioned results revealed that enacting CL requires TRG leaders to be aware of taking on the CL role, possessing skills and knowledge of teaching and management and also professional ethics. In fact, a need exists to improve TRG leaders’ capacity related to enacting the CL role because they are not equipped professionally.

Table 4 summarizes the major findings in relation to TRG leaders’ engagement in CL. With the implementation of the curriculum decentralization, the locus of CL extended to teacher leaders which enables teachers to have more autonomy in taking initiatives at the school level, the classroom level, the social relationship level, and the individual level. However, they still face challenges such as less power in decision making for the national curriculum, less awareness in taking on the leadership role, uncooperative climate among teacher, and lack of knowledge and skills to effectively manage the curriculum.

**Table 4:** Summary of findings related to TRG Leaders’ engagement in CL.

Layer	Major initiatives	Characteristics	Difficulties
School level	Take instructional initiatives	Have more autonomy in classroom teaching for both national curriculum and school-based curriculum (i.e., make teaching arrangement, choose teaching approach, conduct teaching and researching activities)	Cannot make any instructional decision for the national curriculum (i.e., teaching hour, teaching content, textbook selection) No criteria for curriculum evaluations
Classroom level			
Social relationship level	Nurture relations	Communicate with principal, Office of Academic Affairs, and Teaching and Research Center mainly on teaching issues; Nourish peer collaboration; Communicate with stakeholders outside the school	Receive insufficient support from the principals; Get pressure; Face unmotivated climate
Personal level	Promoting individual development	Be experienced in teaching Have ability to perform/lead research; Have future foresight for curriculum development; Have extensive knowledge and experience in teaching.	Lack awareness of taking on the CL role; Be without related knowledge and skills of exercising CL

## Discussion

According to the entire data source extracted from the TRG leaders’ interviews, meeting observations, as well as TRG leaders’ performance summary documents, some insights were confirmed regarding the fulfillment of the CL role.

In terms of the key features, the results fell into three domains. Firstly, it seemed to confirm that no unified definition exists of CL. Evidence from the interviews demonstrated that CL refers to taking instructional initiatives at the classroom level, to the functions and responsibilities for enacting CL, and to the interplay among a set of stakeholders. These findings add to existing evidence about the definitions of CL, that it reflects the conceptions of being responsible for curriculum issues (Wang and Zheng, 2013; Jorgensen, 2016), the functions for school development (Wiles, 2009), and the interrelationships with stakeholders at the social relationship level (Wiles, 2008; Hu and Gu, 2012). However, it is striking to notice that only seven TRG leaders (35%) indicated that they had heard the term. In actual fact, this finding was identified Zhang (2012) in that TRG leaders lacked relevant knowledge of CL and even had not heard of the term. It seems highly reasonable to believe that only since the NCR was implemented in 2001 has research attention been paid to the understanding of CL (Zhang et al., 2014). Moreover, TRG leaders’ unfamiliarity with the term CL is presumably because they have seldom been trained or taught knowledge related to CL role fulfillment. Comparatively, CL is not a new concept in Western studies. This divergence could be explained by the fact the term CL was first presented by Passow in his dissertation “Group-Centered Curriculum Leadership” in 1952, and then became widely recognized by researchers from 1990s (Tsui, 2010; Yin, 2012).

Secondly, a great deal of the commentary revolved around reflections that TRG leaders assume responsibility for curriculum matters, especially regarding the school-based curriculum. In particular, TRG leaders were found to be in charge of formulating the instruction plans, designing teaching schedules and teaching approaches, tailoring teaching materials, and arranging quizzes or exams at the macro-level. Hence, there was evidence in this context at least to support both the Chinese and Western literature’s comments on TRG leaders’ autonomy in the school-based curriculum, that TRG leaders can make decisions for building a holistic view of the curriculum (Macpherson et al., 1996), formulating teaching plans (Handler, 2010; Wang and Zheng, 2013), and selecting instructional materials (Cummings, 2011; Yang, 2012). Moreover, TRG leaders have much autonomy in adjusting the sequence of teaching knowledge points, maintaining an appropriate learning pace for students, and choosing the effective teaching approaches for specific knowledge at the classroom level. This was the first time TRG leaders have described their initiatives in detail, which is disparate from the Chinese literature, in which explanations or descriptions rarely go into any depth about what teachers do during classroom teaching. This result might imply that most Chinese studies focus on exploring the challenges faced by teachers who enact CL (Chang and Li, 2007; Zhang and Fu, 2013), rather than on examining what powers or authorities that they have when empowered. Additionally, the results add to existing evidence about the significance of empowering TRG leaders with autonomy in making decisions for classroom teaching, because it was helpful for improving teaching quality and increasing academic achievements. This resonates with the findings of Luo and Xia (2011) and (Ho, 2010) regarding teachers’ engagement in CL ensuring effective learning and teaching. More speculatively, these TRG leaders are also normal teachers who work in an authentic teaching context, are familiar with students’ diverse learning needs (Huang and Zhu, 2015), and most importantly are “ethically obliged to do whatever is best for their students” (Ho, 2010, p. 614).

Thirdly, all TRG leaders were identified as being in charge of taking teaching and research initiatives after class, such as assessing teaching performance after peer class observations, evaluating students’ learning achievements after tests or examinations, and holding workshops for colleagues to reflect on teaching practice. This result echoes Zheng and Guo’s (2010) claim that enacting CL includes making assessments and evaluations of curriculum and teaching quality. In the same vein, Western researchers have indicated that evaluation initiatives involve conducting regular reviews of students’ learning achievements (Wiles, 2009), writing assessments or reviews of curriculum implementation (Cummings, 2011), or evaluating curriculum activities (Henderson and Hawthorne, 2000).

Although teacher leaders are empowered with decentralized autonomy in taking the CL roles, they encountered challenges in demonstrating CL behaviors. On the one hand, findings revealed that there were two environmental constraints which affect teacher leaders’ initiatives. One, that TRG leaders’ autonomy of taking instructional initiatives was restricted by certain policy regulations (i.e., the national curriculum standards), which leads to the low level of engagement in curriculum matters. This result supports the statement that the initiatives taken for teaching must follow the curriculum standards (Qi, 2011). One reason was that the policy of three-level curriculum management requires the curriculum to be controlled by the central government, local authorities, and schools, respectively, and to be developed in accordance with the national curriculum standards (Feng, 2006). This also resonates with the findings of Western researchers that teachers assume the responsibilities of reviewing and monitoring curriculum policies (Cummings, 2011). Nevertheless, the problems of scant power over the selection of textbooks differ from Western scholars’ claim that teachers are expected to have autonomy in developing teaching resources. Speculatively, the different educational contexts and different educational systems result in dissimilar results, because the social context has effects on teachers’ perceptions and teaching initiatives (Cummings, 2011).

The other is that the environment is uncooperative for TRG leaders to enact the CL role. First, TRG leaders cannot obtain sufficient support from superiors. The results indicated that pressures from principals decreased TRG leaders’ motivation to assume the CL role. This finding is congruent with the statement that there is scant support from principals, although they play a pivotal role in supporting teachers’ initiatives (Chval et al., 2010; Hu and Gu, 2012). Speculatively, the hierarchical schooling system in China results in a particular situation under which teachers enact the leadership role but with less support from their superiors (Lin and Feng, 2007). Moreover, this result echoes the findings of another study (Dong, 2008) regarding the tension between principals and teachers, such as TRG leaders having to follow the principals’ orders, which do not always satisfy teachers’ intentions. Second, the findings revealed that the uncooperative climate and atmosphere among teachers brought difficulties in enacting CL. This is because elderly teachers lack enthusiasm for participating in any activities and young teachers were lazy and procrastinated when taking activities. This result confirms the statement regarding there being little collaboration between leaders and teachers (Xiong and Zhong, 2010). It is striking to notice that the rationales behind the phenomenon of the uncooperative climate are different. This result is disparate from previous research, which found that the teacher performance evaluation system leads to severe competition and an uncooperative climate (Li and Wang, 2010; Fu and Yu, 2014). Unlike the findings of an uncooperative atmosphere among peers in this research, Western research confirmed that teacher leaders are found to be active in interaction and collaboration with peers (Elliott et al., 1999). As Ritchie et al. (2007, p. 151) described, there is a “centrality of successful interactions” among teachers. It is possible that the research contexts in these studies differed from the present research context, which leads to dissimilarities. As Macpherson and Brooker (2000) stated, contextual factors have an influence on enacting CL.

On the other hand, concerning personal situations, this study raises the problem of unmotivated and inactive attitudes held by TRG leaders toward taking on the CL role. In this study, some TRG leaders did not want to take responsibility for curriculum matters, some were unclear about their responsibilities, and some did not want to be empowered. This result provided empirical support for the contention in both Chinese and Western studies that most teacher leaders do not have strong ambitions or desires for assuming the CL role and lack awareness of how to enact CL (Handler, 2010; Ye and Zhu, 2013). This could be explained by teachers having already become used to being followers rather than decision makers (Lu, 2011). As Ho (2010) noted, some teachers are less enthusiastic about making decisions when tasks are imposed by their superiors.

Furthermore, this study indicated that TRG leaders lack the related knowledge, skills, and experience of how to enact the CL role, which caused difficulties for them in managing teachers. The respondents (30%) explained that there is little training related to improving their management skills or communication skills regarding how to enact CL. This result is in line with similar contentions regarding teacher leaders’ insufficient professional knowledge of how to enact CL raised by researchers in this aspect (Xiong and Zhong, 2010). In contrast to this result, teacher leaders in the Western context have been identified as having substantial knowledge and skills of management and communication (Nashashibi and Watters, 2003; Wiles, 2009). This might be because much research attention has been given to understanding CL since the 1990s (Elliott et al., 1999); thus, teachers do not lack knowledge related to CL.

Moreover, it was striking to notice that although 18 TRG leaders (90%) confirmed the importance of possessing professional ethics for engaging in CL, TRG leaders were found to lack professional ethics when taking on the CL role. For example, they were identified as being irresponsible, aggressive, selfish, and not persistent when facing difficulties. This point is quite similar to that of Lin and Feng (2007) as well as Ye and Zhu (2013), who asserted that teachers leaders lack a sense of responsibility, and are also selfish. Unlike Chinese studies, such low levels of professional ethics cannot be found in Western literature. This divergence of having moral literacy could be explained by such issues as professional ethics having been discussed and criticized by theorists and practitioners since 1915 in the Western educational context (Campbell, 2000). This could partly explain why professional ethics is an immature area in Chinese research.

## Conclusion

This study explored the conceptions of how TRG leaders engage in CL in the Chinese context. Overall, the results demonstrated that the curriculum decentralization empowered teacher leaders with more autonomy in taking instructional initiatives, echoing studies suggesting that decentralization enables teachers to have more democratic participation in making decisions for school and curriculum matters (Ho, 2005; Law et al., 2010). Thus, this result is in accord with the international trend toward curriculum decentralization, and highlights the importance of empowering TRG leaders with the autonomy to make curriculum decisions.

More importantly, the current research provides empirical data for further understanding how TRG leaders take initiatives to enact the CL role. As addressed in the literature, few studies have investigated CL using empirical data, and the majority of these are commentary studies that have drawn conclusions and arguments without any concrete empirical data (Hu and Gu, 2012). Thus, this study also adds to those Chinese studies that provide solutions to problems emanating from Western studies and to the growing body of literature on teachers’ engagement in CL. Last but not the least, the results may be of great use to principals for realizing to what extent autonomy should be devolved to teacher leaders and how to support teacher leaders to exercise leadership, and to teacher leaders’ for being resilience in performing their CL leadership role.

Although these findings are encouraging for CL research, the present study has some limitations. The sample comprised only 10 secondary schools, making it an unrepresentative sample of secondary schools in the Chinese context. Future research may aim to conduct similar research in other contexts with large-scale samples and obtain an enhanced understanding of its multi-faceted nature, which will ultimately contribute to enhancing the current understanding of CL in the international domain.

## Data availability statement

The raw data supporting the conclusions of this article will be made available by the authors, without undue reservation.

## Ethics statement

The studies involving human participants were reviewed and approved by Faculty Human Research Ethics Committee, The Education University of Hong Kong. The patients/participants provided their written informed consent to participate in this study. Written informed consent was obtained from the individual(s) for the publication of any potentially identifiable images or data included in this article.

## Author contributions

YS conceived the original idea of this paper. This was also discussed with JC. The manuscript was written by YS which involves collecting and analyzing the data. JC gave a significant help in drafting the manuscript with many helpful suggestion. All authors contributed to the article and approved the submitted version.

## Funding

This work was funded by Guangdong Planning Office of Philosophy and Social Science (GD21YJY08).

## Conflict of interest

The authors declare that the research was conducted in the absence of any commercial or financial relationships that could be construed as a potential conflict of interest.

## Publisher’s note

All claims expressed in this article are solely those of the authors and do not necessarily represent those of their affiliated organizations, or those of the publisher, the editors and the reviewers. Any product that may be evaluated in this article, or claim that may be made by its manufacturer, is not guaranteed or endorsed by the publisher.
